# Downstream interaction by glucagon-like peptide-1 and glucose-dependent insulinotropic polypeptide agonism is required for synergistic effects on body weight

**DOI:** 10.1016/j.molmet.2025.102214

**Published:** 2025-07-16

**Authors:** Claire H. Feetham, Minrong Ai, Isabella Culotta, Alessia Costa, Jenna Hunter, Robert A. Brown, Tamer Coskun, Paul J. Emmerson, Giuseppe D'Agostino, Simon M. Luckman

**Affiliations:** 1 Faculty of Biology, Medicine and Health, University of Manchester, Manchester, UK; 2 Lilly Research Laboratories, Eli Lilly & Company, Indianapolis, IN, United States

**Keywords:** Glucagon-like peptide-1, Glucose-dependent insulinotropic polypeptide, Dual-incretin agonist, Obesity, Tirzepatide

## Abstract

**Objectives:**

Dual glucagon-like peptide-1 receptor and glucose-dependent insulinotropic polypeptide receptor agonists (GLP1RA and GIPRA, respectively) synergise to reduce body weight. Though this synergy depends on receptors within the brain, where and how this occurs is unclear.

**Methods:**

We employed a combination of neuroanatomical approaches in the mouse to investigate access of the dual GLP1RA/GIPRA, tirzepatide, and study the central targets engaged by single agonist, dual agonist and combined agonist treatments. Genetic manipulations were then used to further investigate the functional significance of specific brain regions and distinct neuronal subtypes.

**Results:**

We recorded penetration of fluorescently labelled tirzepatide limited mainly to circumventricular organs and confirmed the importance both GLP1R and GIPR in the dorsal vagal complex for the actions of systemically administered agonists. Receptor expression indicates GIPRA alone activates a distinct population of GABA neurons in the area postrema directly, but also neurotensin neurons in the central amygdala (Nts^CeA^) indirectly. Disabling Nts^CeA^ neurons selectively reduces the synergistic effect of dual GLP1R/GIPR agonist administration on body weight.

**Conclusions:**

As with selective GLP1RA, the actions of dual GLP1RA/GIPA appear to be dependent on the dorsal vagal complex for their action, probably most importantly by gaining access through the area postrema. Downstream targets include the central amygdala where signals following dual receptor agonism interact. Specifically, Nts^CeA^ neurons are required for the full synergistic effect of dual receptor agonism on body weight.

## Highlights

• Tirzepatide accesses the brain at circumventricular organs.
• Systemic incretin receptor agonists depend on brainstem receptors for weight-reducing effects.
• Glucose-dependent insulinotropic polypeptide agonism activates distinct neuron populations.
• Central amygdala neurotensin neurons are required for incretin receptor agonist synergy.

## 1 Introduction

Long-acting, glucagon-like peptide 1 receptor agonists (GLP1RA), such as semaglutide (SEMA) and dulaglutide, are effective treatments which cause weight loss primarily by reducing appetite [[1], [2], [3]]. They are also safe and well tolerated drugs; though some patients report gastrointestinal adverse effects, notably nausea at least at the beginning of treatment, which can lead to long-term non-adherence [4]. Up-titration of GLP1RA may reduce the gastrointestinal effects experienced by patients [5,6]. Single molecule dual agonists that have affinity at both GLP-1 and glucose-dependent insulinotropic polypeptide (GIP) receptors, such as tirzepatide (TZP) and the preclinical compound MAR709, have improved efficacy when compared with mono-GLP1RA -based drugs [[7], [8], [9], [10], [11]]. Importantly, TZP (Mounjaro®) is effective at causing weight loss, and may have relatively better GI tolerability compared with selective GLP-1RA. For example, preclinical models showed reduced aversive behaviour, which has been explained by the co-incident inhibition of neurons in the brainstem area postrema (AP) that co-localise GLP1R, cholecystokinin (CCK) and GDNF-family receptor α-like (GFRAL), the receptor for the cytokine, growth differentiation factor 15 (GDF15) [[12], [13], [14]]. Removing adverse effects may allow higher clinical dosing and greater efficacy. However, since improved efficacy is also observed in preclinical models, it is unlikely that better compliance alone can explain the enhanced efficacy. Thus, the apparent synergistic effect between GLP1R and GIPR to cause greater weight loss than GLP1R mono-agonists alone [15,16] and how this is achieved remains unclear [17,18].

GLP1R are widely expressed in the mammalian brain and normally respond to GLP-1 produced by preproglucagon (PPG; encoded by the *Gcg* gene) neurons in the caudal brainstem rather than GLP-1 produced in the gut, since the latter is rapidly broken down in the circulation by dipeptidyl peptidase [19]. By contrast, clinically administered GLP1RA have been developed to increase half-life, and to reach much higher effective concentrations in the circulation [1,10]. Even so, these drugs have limited brain penetration, mostly limited to circumventricular organs (CVOs) or adjacent, underlying structures [[20], [21], [22]]. The primary site(s) of action have not been fully elucidated, though the consensus has been that they have a distributed effect (e.g. at the hypothalamic arcuate nucleus; ARH) and/or brainstem [21,23]. However, recent papers suggest that the weight-reducing effects of systemic GLP1RA are dependent primarily on the brainstem rather than the hypothalamus [13,24,25]. The distribution of GIPR is less well documented. Published histological analysis of *Gipr* mRNA or binding suggest its distribution is very limited in the rodent brain [26,27]. This contrasts with more recent studies using a *Gipr*-Cre reporter mouse, which have described widespread expression of the transgene in the adult mouse brain [28,29].

Work from our laboratory and others has sought to identify populations of neuron in the AP, the adjacent nucleus tractus of the solitarius (NTS) and downstream structures involved in the effects of circulating incretin receptor agonists [23,25,[30], [31], [32], [33]]. As noted, a sub-population of CCK neurons in the AP and adjacent NTS, are necessary for the full anorectic, body weight-reducing and aversive effects of both the GLP1RA, exendin-4 (EX4), and GDF15 [[12], [13], [14]]. This population of GLP1R/CCK/GFRAL cells project to the exterolateral parabrachial nucleus (PBN), where they engage a well-characterised pathway mediating aversive responses [12,34], as well as to the underlying NTS [12,25]. The activation of GLP1R/CCK/GFRAL neurons by EX4 is diminished by co-administration of the stable GIPRA, [D-Ala^2^]-GIP, due to stimulation of adjacent GABAergic cells that express GIPR [13,14]. Importantly, GIPR agonism in the brainstem blocked the aversion, but not acute anorexia caused by EX4 [13], and global knock-down of *Gipr* in GABAergic neurons blocks the synergistic effect of dual-receptor agonism [[35], [36], [37]]. Together, these studies have provided insight into central targets for incretin drugs but have yet to fully explain the pathways leading to increased efficacy of dual agonist drugs on weight loss. Here we provide evidence that TZP can access the brain at all CVOs. However, as described previously [13,24], the weight-reducing effect of GLP1R agonism appears to be primarily dependent on its action in the brainstem. Functional activity mapping using single receptor agonism or combinations identified neurotensin (*Nts*)-expressing neurons in the central amygdala (CeA) as a distinct molecularly defined neuronal population recruited by GIPRA. Disabling these neurons attenuated the effects of dual receptor agonism on body weight, providing the first description of a central cellular substrate where GLP1RA and GIPRA signals intersect to cause synergistic effects on body weight.

## 2 Methods

### 2.1 Animals

*Nts*^Cre^ (B6;129-Nts^tm1(cre)Mgmj^/J, stock # 017525), *Gad2*-IRES-Cre (Gad2^tm2(cre)Zjh^/J, stock # 010802) and R26R-EYFP (B6;129X1-Gt(ROSA)26Sor^tm1(EYFP)Cos^/J, stock # 006148) mice (all Jackson Laboratories, ME, USA) were bred in house. *Glp1r*^wt/wt^ and *Glp1r*^flox/flox^ mice were rederived from frozen sperm (kindly gifted by Dr Jon Campbell, Duke University, NC, USA) [38] and bred in house. C57BL/6J mice were obtained from Charles River (Manston, Kent, UK), Taconic (NY, USA) or Janvier (Laval, France). All mice were group housed unless otherwise stated and kept on a 12:12 h light:dark cycle, at room temperature (22 ± 2 °C; 50–55% humidity). Mice were provided with *ad libitum* access to water and standard rodent chow (#801151 RM1-P; Special Diet Services, Witham, Essex, UK or Altromin 1324, Brogaarden, Denmark) unless otherwise stated. All animal studies were performed in accordance with the UK Animals and Scientific Procedure Act, 1986, and approved by the local ethics committee and in compliance with Eli Lilly and Company's Institutional Animal Care and Use Committee.

### 2.2 Drugs and viral vectors

TZP was fluorescently labelled with AFDye 647 Azide at its C terminus, as previously described and shown to maintain its functional potency at GLP1R and GIPR through cAMP accumulation assays [39,40]. TZP-AF647 (30 nmol kg^−1^; 5 ml kg^−1^; subcutaneous, SC), TZP (30 nmol kg^−1^; 5 ml kg^−1^; SC), semaglutide (SEMA; 30 nmol kg^−1^; 5 ml kg^−1^; SC), [D-Ala^2^]-GIP (100 μg kg^−1^; 10 ml kg^−1^; intraperitoneal, IP), GLP-140 (10 nmol kg^−1^ or 30 nmol kg^−1^; 10 ml kg^−1^; SC) and GIPFA-085 (300 nmol kg^−1^; 10 ml kg^−1^; SC) were supplied by Eli Lilly (Indianapolis, USA) and dissolved in vehicle (Tris–HCl 8.0 pH 40 mM and 0.02% Tween 80 made up in ddH_2_O). Clozapine N-oxide (CNO; Cat.# 4936; Tocris Bioscience, Bristol, UK) was dissolved in saline (1 mg kg^−1^; 4 ml kg^−1^; IP).

For NTS injections, *Glp1*r^wt/wt^ and *Glp1r*^flox/flox^ male mice were injected using a modified stereotaxic injection procedure as previously described [41]. Briefly, mice were anaesthetized with a mixture of ketamine and xylazine dissolved in saline (80 and 10 mg kg^−1^, respectively; 10 ml kg^−1^; IP). Mice were placed in a stereotaxic frame, an incision was made at the level of the *cisterna magna*, and neck muscles were carefully retracted. Following an incision in the *dura*, the *obex* served as a reference point for injections. Mice were injected bilaterally with AAV9-hSyn-Cre-P2A-TdTomato (titre: 1 x 10^12^ gc ml^-1^; Cat.# 107738-AAV9; Dr Rylan Larsen, Addgene, MA, USA) using a glass micropipette attached to a Nanoject II Auto Nanoliter Injector (Drummond Scientific Company, PA, USA). NTS coordinates were approximately 0.2 mm A/P, ±0.2 mm M/L, −0.2 mm D/V from the *obex*. All animals were administered analgesia (5 mg kg^−1^; SC; Carprofen; Pfizer, NY, USA) for 2 days post-operatively.

For all other intercranial injections, 8-week-old male and female mice were anaesthetised with 2–3% isoflurane in oxygen and administered analgesia (0.1 mg kg^−1^; SC; buprenorphine; Animalcare, York, UK) before the start of surgery. Mice were placed in a stereotaxic frame, the skull was exposed and a small hole drilled above the injection site. All co-ordinates were determined using the Mouse Brain Atlas [42]. Viral vectors and tracers were injected using a Nanoject II automatic nanolitre injector fitted with a pulled glass micropipette (Drummond Scientific Company).

For neuronal silencing studies, AAV-DJ-CMV-DIO-eGFP-2A-TeNT (titre: 1 x 10^12^ gc ml^-1^; Cat.# AAV-71; Stanford Vector and Viral Core; Stanford University, USA) or control AAV8-hSyn-DIO-mCherry (titre: 1 x 10^12^ gc ml^-1^; Cat.# 50459-AAV8; Dr Bryan Roth, Addgene) were injected bilaterally into the CeA of *Nts*^Cre^ mice (20–30 weeks old) at a total volume of 120 nl per side (coordinates from bregma: −1.2 mm A/P, −2.4 mm M/L, −5.1 mm D/V). For neuronal activation studies in *Nts*^Cre::EYFP^ mice (20–30 weeks old) were injected bilaterally into the CeA, with AAV8-hsyn-DIO-hM3Dq-mCherry (titre: 2.2 x 10^12^ gc ml^-1^; Cat.# 44361-AAV8; Dr Bryan Roth, Addgene) in a total volume of 200 nl per side. For anterograde tracing studies, AAV8-hSyn-DIO-ChR2(h134r)-mCherry (titre: 1.9 x 10^13^ gc ml^-1^; Cat.# 20297-AAV8; Karl Deisseroth; Addgene) was injected unilaterally into the CeA of *Nts*^Cre^ mice in a total volume of 120 nl.

For retrograde tracing studies, FluoroGold (hydroxystilbamidine, 4% w/v solution in water; Invitrogen, ThermoFisher, Massachusetts, USA), was injected unilaterally into the PBN of male and female *Gad2*^Cre::EYFP^ mice at a total volume of 150 nl (coordinates from bregma: −5.0 mm A/P, −1.5 mm M/L, −4.0 mm D/V). After allowing two weeks for recovery and axonal transport, mice were perfused transcardially (see below).

At the end of each surgery, all wounds were sutured, animals were provided post-operative care and left to recover for at least 2 weeks before experimentation.

### 2.3 Fluorescent TZP

Fluorescent TZP studies were performed at Gubra (Horshom, Denmark). C57BL/6J male mice (9–10 weeks old) were singly housed and handled for two weeks prior to the start of the experiment. On the day of the study, mice were injected with vehicle or TZP-AF647. Two hours later, the mice were deeply anaesthetised with 4–5% isoflurane in oxygen and transcardially perfused with phosphate-buffered saline (PBS) followed by 10% neutral-buffered formalin. Brains there then dissected and immersion fixed overnight at room temperature. Tissue clearing was performed using a standard iDISCO (immunolabeling-enabled three-dimensional imaging of solvent cleared organs) protocol [43]. Briefly, the samples were washed three times for 30 min in PBS and dehydrated in a methanol/H_2_O gradient (20%, 40%, 60%, 80% and 100%) for 1 h each at room temperature, followed by an overnight incubation in 100% methanol. The following day they were incubated in 66% dichloromethane/33% methanol for 3 h at room temperature and washed in 100% dichloromethane. Samples were then stored in dibenzyl ether in the dark until imaging.

Samples were imaged using a LaVision Ultramicroscope system II (Miltenyi Biotec Ltd, Surrey, UK) and an Olympus MV PLAPO 2X C objective (Olympus, Tokyo, Japan) at 1.26× magnification. Fluorescent signal was captured at autofluorescence channel (560 nm) and at compound specific channel (647 nm). Dibenzyl ether was used as clearing agent during acquisition of data. Aivia software (Leica Microsystems Ltd, Milton Keynes, UK) was used for 3D visualization of data and customised software was used for image analyses (Gubra).

### 2.4 Whole-brain FOS

Whole-brain FOS was performed at Gubra. C57BL/6J male mice (9–10 weeks old) were singly housed, handled for two weeks and given subcutaneous injections of vehicle PBS for 4 days prior to the start of the experiment. On the day of the study food was removed immediately prior to dosing and mice were injected with vehicle, SEMA or TZP 2 h before perfusion fixation (as above). Whole brain clearing was performed using the standard iDISCO protocol. Following dehydration, samples were bleached in chilled 5% hydrogen peroxide overnight at 4 °C. Samples were subsequently rehydrated and washed in PBS with 0.2% Triton X-100 (PBS-T). Samples were then incubated in permeabilisation solution (PBS-T containing glycine, 25% dimethyl sulfoxide, 0.02% sodium azide) at 37 °C for 3 days and incubated in blocking solution (6% donkey serum) at 37 °C for 2 days. Following blocking, the samples were incubated with rabbit anti-cFos (Cat.#2250, Cell Signalling Technology, MA, USA) in PBS-T and heparin/5% dimethyl sulfoxide/3% donkey serum at 37 °C for 7 days. They were then washed and incubated with donkey anti-rabbit Cy5 (Cat.#711-175-152; Jackson ImmunoResearch, PA, USA) in 3% donkey serum at 37 °C for 7 days, followed by further washes.

Brain samples were imaged using a Lavision ultramicroscope system (Miltenyi Biotec Ltd) and MV PLAPO 2XC objective (Olympus) or Bruker LCS SPIM (Bruker, MA, USA). Ethyl-3-phenylprop-2-enoate was used as a clearing agent during the acquisition of data. Aivia software (Leica Microsystems Ltd) was used for 3D visualization of data and customised software was used for all image analyses (Gubra). For Z-score heatmap images, coronal sections were taken from the Gubra brain atlas, upon which “density heatmaps” of group averages were superimposed. The heatmaps depict the group-average density of detected FOS cells (within a 100 μm radius) at each voxel position. Regions displaying large and consistent differences in the number of FOS positive cells (Z-score >1.96, corresponding to p < 0.05 (uncorrected)) were visualized in glow scale. Voxel-level statistics are only calculated in regions with a density of >3 cells in a 100 μm radius. Brighter colours on a continuous scale illustrate a higher Z-score (larger consistent differences between the average FOS signal in the analysed groups). For each region, a negative binomial generalized linear model was fitted to the cell count data, and a subsequent Dunnett's test performed for multiple comparisons. All significantly regulated regions go through statistical validation to see if they align with the assumptions of normality and homoscedasticity. These areas undergo further validation to ensure the quantified signal originates from the region of interest and does not spill over from a neighbouring region. Top brain regions were selected by fitting data to a one-factor linear regression model with the treatment groups as categorical, independent (predictor) variables. Validated areas were then either tested for statistical significance against the control treatment using a Student's paired t-test or multiple treatments were compared using ANOVA with appropriate *post hoc* tests.

### 2.5 *Gipr* and *Glp1r* RNAscope

12-week-old male mice and rats were culled and dissected brains and pancreata frozen immediately on dry ice. Sections of the pancreas were used to validate the probes (Suppl Fig. 3A). RNAscope *in situ* hybridization for mouse *Gipr* (Cat.# 319121, Advanced Cell Diagnostics, CA, USA), mouse *Glp1r* (Cat.# 418851), rat *Gipr* (Cat.# 318801) and rat *Glp1r* (Cat.# 315221) was performed on 8 μm cryo-sections covering the brainstem and hypothalamus according to manufacturer's instructions (Advanced Cell Diagnostics) at Gubra. After hybridization, sections were counterstained with DAPI and coverslipped using a fluorescence mounting medium. Slides were scanned under a 20x objective in an Olympus VS-120 slide scanner (Olympus Corporation, Tokyo, Japan) with appropriate fluorescent filters.

### 2.6 Tissue preparation and immunohistochemistry on free-floating sections

For additional neuronal activation FOS studies, male and female mice were administered treatments 2 h before transcardial perfusion. For all immunohistochemistry on free-floating sections, mice were deeply anaesthetised with 4–5% isoflurane in oxygen and transcardially perfused with heparinsed saline (2 kU l^−1^, Sigma-Aldrich, Welwyn, UK) followed by 4% paraformaldehyde made up in 0.1 M phosphate buffer (Sigma-Aldrich). Brains were carefully dissected and post-fixed in 4% PFA overnight, followed by cryoprotection in 30% sucrose at 4 °C. Brains were cut into 30 μm-thick coronal sections using a freezing sledge microtome (Bright 8000, Cambridge, UK) and either processed immediately or stored in cryoprotectant solution at −20 °C. Sections were washed in PB-T and blocked in 5% normal serum for 1 h. Following blocking, sections were incubated in primary antibody made up in 1% normal serum in PB-T for 20 min at room temperature, then overnight at 4 °C. The next day, sections were washed in 0.1 M PB and then incubated in secondary antibody (in 5% normal serum) for 2 h. Finally, sections were washed in 0.1 M PB followed by water, mounted onto glass slides, air-dried overnight and coverslipped with Prolong Gold (Molecular Probes, Eugene, OR, USA).

Primary antibodies used were rabbit anti-cFos (Cat. #190289, Abcam, Cambridge, UK); guinea pig anti-neurotensin (Cat. #418005, Synaptic Systems, Goettingen, Germany); chicken anti-GFP (Cat. #AB13970, Abcam); rabbit anti-DsRed (#632496; Takara Bio Europe, Saint-Germain-en-Laye, France); goat anti-mCherry (#AB0040-200, Sicgen, Cantanhede, Portugal). Secondary antibodies used were donkey anti-chicken Alexa Fluor 488 (Cat.# 703-545-155, Jackson ImmunoResearch), donkey anti-rabbit Alexa Fluor 488 (Cat.# 711-545-152, Jackson ImmunoResearch), donkey anti-rabbit Alexa Fluor 594 (Cat.# 711-585-152, Jackson ImmunoResearch).

Images were acquired on a 3D-Histech Panoramic-250 microscope slide scanner using a 20x/0.80 Plan Apochromat objective (Zeiss, Birmingham, UK) and the FITC and Texas Red filter sets. Snapshots of the slide scans were taken using the SlideViewer software (3D-Histech, Budapest, Hungary). Further images were collected on a Zeiss Axioimager.M2 upright microscope using a 5x or 10x Plan Apochromat objective and captured using a Coolsnap HQ2 camera (Photometrics, Harpenden, UK) through Micromanager software v1.4.23. Specific band pass filter sets for DAPI, FITC and Texas Red were used to prevent bleed through from one channel to the next. Images were processed and analysed using Fiji ImageJ (http://imagej.net/Fiji/Downloads). For FOS quantification sections were counted blind and manually and averaged per section.

### 2.7 Feeding studies

To induce diet-induced obesity (DIO), mice were given high-energy diet (HED; # 824054 60% energy from fat; Special Diet Services, Witham, UK) for a minimum of 6 weeks prior to the start of the experiment. Mice were weighed weekly to ensure weight gain, mice with less than a 10% increase in body weight were excluded. This dietary regimen resulted in DIO body weights of around 35–45 g at study start.

For all chronic feeding studies mice were initially group housed before habituation to single housing and handling for a minimum of one week prior to the start of the experiment. After acclimation, body weight and food intake were recorded manually at the same time each day for at least 4 days prior to the start of treatment. For neuronal silencing studies *Nts*^Cre:CeA−TeNT^ mice were singly housed for a minimum of one week before being put into an automated feeding system (Sable Systems International, NV, USA). Body weight was measured daily at the same time each day.

### 2.8 Statistics

Details of statistical analyses are provided in the corresponding figure legends and were carried out using Prism statistical package (GraphPad Software Inc, San Diego, USA). Data are displayed as mean ± SEM. Student's t-tests were used to analyse two experimental groups and multiple groups were analysed using ANOVA with appropriate *post hoc* tests. P values less than 0.05 considered as statistically different and denoted as follows: ∗p < 0.05, ∗∗p < 0.01, ∗∗∗p < 0.001 and ∗∗∗∗p < 0.0001.

## 3 Results and discussion

### 3.1 TZP can access the brain

**Figure 1:**
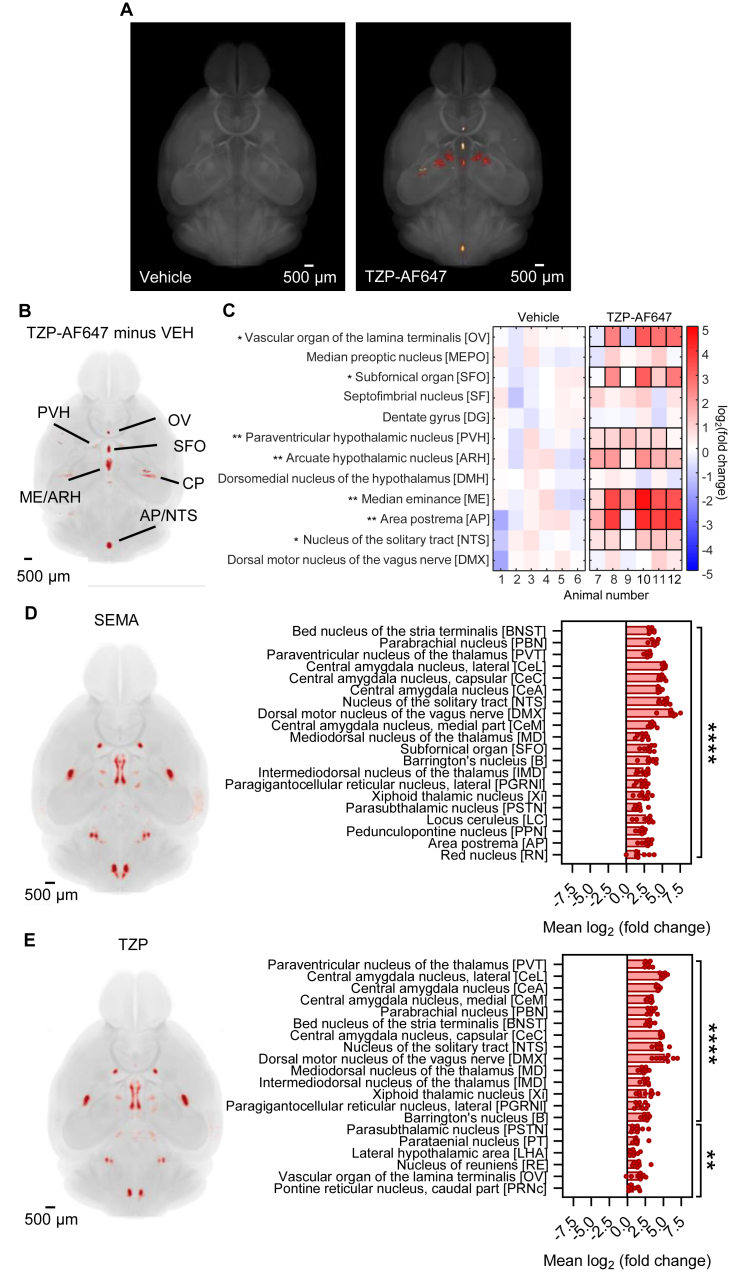
**TZP-AF647 accesses the brain.** (A) Overview of the average fluorescence across mice administered with vehicle or TZP-AF647 (30 nmol kg^−1^; n = 6 in each group). The TZP-AF647 fluorescent signal is shown in glow, and the brain background autofluorescence is grey. (B) TZP-AF647 induced fluorescence compared with the vehicle group. (C) Brain regions where TZP-AF647 fluorescence is increased compared to the mean of the vehicle group are shown in red, and areas with a reduced fluorescence in the TZP-AF647 group compared to the vehicle are shown in blue (mean log_2_ (fold changes)). Areas showing statistically significant fluorescence intensity changes are highlighted by black outlines (∗p < 0.05, ∗∗p < 0.01. Student's unpaired t-test). (D) Changes in FOS activity in SEMA (30 nmol kg^−1^) and (E) TZP (30 nmol kg^−1^) treated mice compared to mice administered with vehicle ((mean log_2_ (fold change)); n = 8 per group; ∗∗p < 0.01, ∗∗∗∗p < 0.0001, Students unpaired t-test). Brain regions where FOS activity is increased in the treatment group compared to vehicle are shown in red. Scale bars are 500 μm. Data are presented as mean ± SEM. AP, area postrema; ARH, arcuate hypothalamic nucleus; CP, caudoputamen; ME, median eminence; NTS, nucleus of the solitary tract; OV, vascular organ of the lamina terminalis; PVH, paraventricular hypothalamic nucleus; SFO, subfornical organ.

To determine where TZP accesses the brain, mice were given a single, subcutaneous injection of fluorescent TZP-AF647 (30 nmol kg^−1^) and brains collected 2 h later. TZP-AF647 entry to the brain was visualised in major blood vessels, choroid plexus and CVOs (Suppl Video 1). Background fluorescence signal measured after injection of vehicle, was subtracted to allow an unbiased evaluation of which brain structures are accessed (Figure 1A–C). CVOs in the forebrain (organum vasculosum of the lamina terminalis, OVLT; subfornical organ, SFO; median eminence, ME) and hindbrain AP lack a blood–brain barrier and have open access to circulating TZP-AF647. A strong signal was also visualised in the hypothalamic ARH and the NTS, which lie adjacent to the ME and AP, respectively, and are often considered as having an incomplete blood–brain barrier. Interestingly, there was also a strong signal in the paraventricular nucleus of the hypothalamus (PVH; but not in ventromedial, dorsomedial or supraoptic nuclei; Figure 1B). Fluorescent TZP produced comparable results as reported for SEMA, liraglutide or LUXendin645 [[20], [21], [22]]. The implication is that clinically important incretin receptor agonists are effective with systemic administrations, even though they may not cross the blood–brain barrier into deeper structures.

Supplementary video related to this article can be found at https://doi.org/10.1016/j.molmet.2025.102214

The following is the supplementary data related to this article:

Multimedia component 2 Multimedia component 2

To determine the functional significance of this brain penetration, we carried out a whole-brain, automated analysis of neuronal activity induced following injection of vehicle (VEH), SEMA (30 nmol kg^−1^) or TZP (30 nmol kg^−1^) (Suppl Videos 2–4). Immunohistochemistry for FOS protein was performed on iDISCO-cleared brains followed by light-sheet microscopy and brain regions assessed automatically. The pattern of activity, following subtraction of VEH, was mostly the same between SEM and TZP: unbiased measurement of the twenty brain regions showing the largest statistical differences in the number of cells expressing FOS are provided for each treatment (Figure 1D,E). A more systematic analysis of areas of interest indicated both SEMA and TZP induced a significant increase in the number of FOS-positive neurons in the NTS, dorsal motor nucleus of the vagus (DMX), PBN, the bed nucleus of the stria terminalis (BNST) and the central amygdala nucleus (CeA; Suppl Fig. 1). The level of activation caused by TZP was lower statistically in the AP, NTS, PBN and CeA. SEMA, but not TZP, also caused an increase in the ARH, ventral tegmental area (VTA) and the parasubthalamic nucleus (PSTN). TZP, but not SEMA, caused a statistical increase in FOS in the lateral hypothalamic area (LHA). The overall pattern of activity is similar with that reported for other, single agonist GLP1RA [21,44] with the caveat that small and/or adjacent anatomical regions may not always be picked accurately during whole-brain imaging procedures. While we cannot rule out direct activation of deep brain structures that possess GLP1R, such as the PBN, CeA and VTA, the more likely explanation for their increased FOS expression is that these structures are downstream of CVOs and their adjacent structures and are activated as a result of network activity.

Supplementary video related to this article can be found at https://doi.org/10.1016/j.molmet.2025.102214

The following are the supplementary data related to this article:

Multimedia component 3 Multimedia component 3

Multimedia component 4 Multimedia component 4

Multimedia component 5 Multimedia component 5

### 3.2 Comparison of brain activity by a long-acting GLP1RA and/or a selective GIPRA

**Figure 2:**
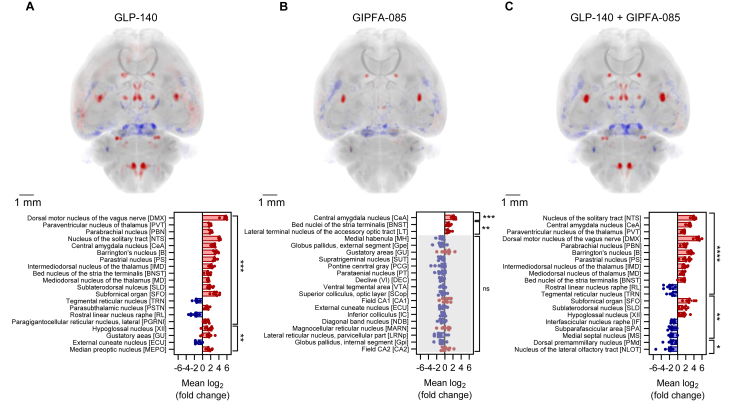
**GLP1 and GIP receptor agonists alone and in combination induce differing neuronal activation patterns.** (A) Changes in FOS activity between mice treated with GLP-140 (30 nmol kg^−1^), (B) GIPFA-085 (300 nmol kg^−1^) or (C) GLP-140 and GIPFA-085 in combination (n = 8 per group) compared to vehicle (n = 7) treated mice (mean log_2_ (fold change)); ∗p < 0.05, ∗∗p < 0.01, ∗∗∗p < 0.001, ∗∗∗∗p < 0.0001, Student's unpaired t-test). Brain regions where FOS activity is increased in the treatment group compared to vehicle are shown in red, and areas with reduced FOS activity in blue. Scale bars are 1 mm. Data are presented as mean ± SEM.

As we wished to distinguish the contributions of GLP1R versus GIPR on the activity pattern elicited by TZP, we did a similar unbiased, automated survey of brain activation with mice given SC injections of VEH, a long-acting GIPR agonist (GIPFA-085) [16], a long-acting GLP1R agonist (GLP-140) or the two agonists together. Each treatment was compared with vehicle so that it was possible to also note areas where FOS activity was reduced. GLP-140 or the two agonists together caused significant induction of FOS in the SFO, OVLT/median preoptic, PSTN, CeA, BNST and midline thalamic structures (Figure 2A–C). Within the brainstem, strong FOS induction was noted in the PBN, NTS, DMX and the hypoglossal nucleus (XII) in the GLP-140 alone and combination groups (Suppl Figs. 2B, C, F, G, I). No significant increases in FOS were recorded in hypothalamic structures in this experiment, which contrasts with the experiment above. Interestingly, the only brain regions to show a significant FOS response to GIPFA-085 alone are the CeA and, to a lesser extent the BNST (Suppl Fig. 2A). These two regions of interest were re-analysed more closely, and FOS counting indicated that the CeA was activated significantly by GIPFA-085 and GLP-140 alone, as well as in the combination group (Suppl Figs. 2A, D, E).

### 3.3 Repeat injection of long-acting GLP1RA reduce body weight through the brainstem

**Figure 3:**
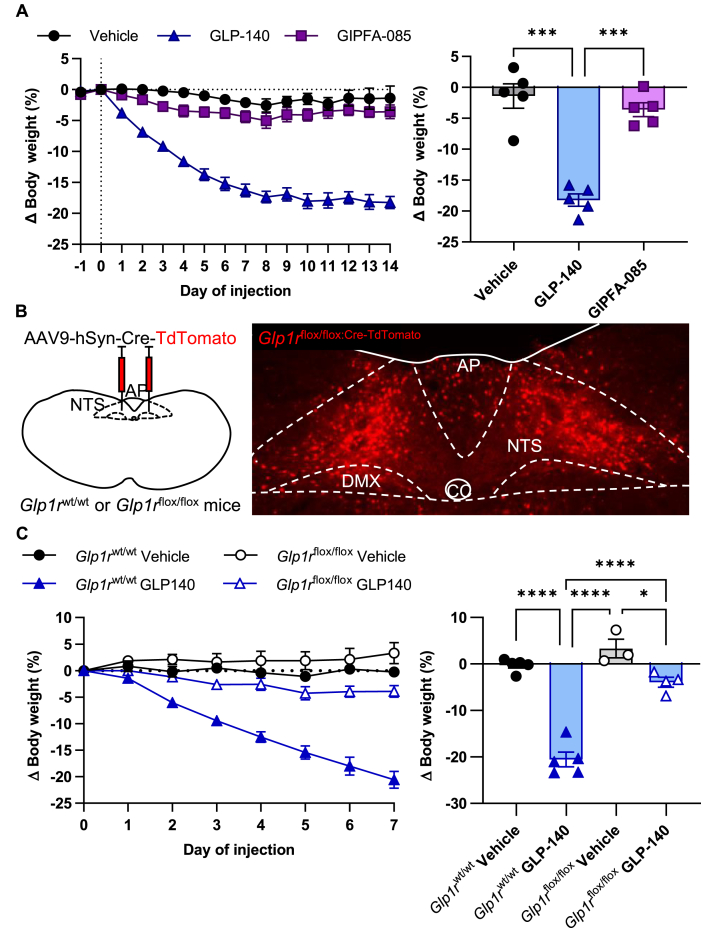
**Glp1r^AP/^^NTS^ neurons are required for the body weight loss effects of GLP-140.** (A) Daily subcutaneous injection of, GLP-140 (blue triangles), but not GIPFA-085 (purple squares), causes a reduction in body weight compared to vehicle (black circles; 30 nmol kg^−1^ and 300 nmol kg^−1^, respectively; n = 5 per group; ∗∗∗p < 0.001 by two-way ANOVA followed by Tukey's *post hoc* multiple comparison test). (B) Schematic illustrating bilateral injection of AAV9-hSyn-Cre-TdTomato into the brainstem of *Glpr1r*^wt/wt^ or *Glpr1r*^flox/flox^ mice (left panel). Representative immunohistochemical image of AAV9-hSyn-Cre-TdTomato (red) into the AP/NTS of *Glpr1r*^flox/flox^ (*Glp1r*^flox/flox:Cre−TdTomato^) mice (right panel). Scale bar is 100 μm. (C) Body weight is significantly reduced in *Glpr1r*^wt/wt^ mice following daily treatment with GLP-140 (blue closed triangles) compared to vehicle (black closed circles; n = 5 per group), but not in *Glp1r*^flox/flox:Cre−TdTomato^ mice (vehicle, black open circles, n = 3, GLP-140, blue open triangles, n = 4; ∗p < 0.05, ∗∗∗∗p < 0.0001, two-way ANOVA followed by Tukey's *post hoc* multiple comparison test). Data are presented as mean ± SEM. AP, area postrema; CC, central canal; DMX, dorsal motor nucleus of the vagus nerve; NTS, nucleus of the solitary tract.

We gave daily doses of the long-acting GIPFA-085 or GLP-140 for 14 days to DIO wild-type C57BL/6J mice and measured food intake and body weight (Suppl Fig. 3A; Figure 3A). Confirming the literature [7,15,16], in our hands GLP1RA treatment reduces body weight by approximately 20%, whereas GIPRA has no significant effect.

There is a body of evidence that suggests the hypothalamic ARH is required for the actions of systemically administered GLP1RA [21]. However, previously, we have shown that disabling brainstem neurons blocks the effects of repeated dosing of EX4 [13]. Likewise, a more recent paper found that longer-term actions of SEMA are blocked if GLP1R are knocked down in the AP/NTS rather than in the ARH [24]. We asked if the longer-term effects of GLP-140 in obese animals is equally dependent on brainstem receptors. We injected AAV-hSyn-Cre-TdTomato into the AP/NTS of homozygous *Glp1r*^flox/flox^ mice or wild-type *Glp1r*^wt/wt^ littermates (Figure 3B). Mice were then placed on high-energy diet to induce obesity. Daily dosing of DIO *Glp1r*^wt/wt^ mice with GLP-140 caused the predicted 20% reduction in body weight (Figure 3C), accompanied by anorexia (Suppl Fig. 3B). These effects were lost completely in *Glp1r*^flox/flox^ mice. This result, with those published previously by us and others [13,24], strongly implicate receptors in the AP/NTS as being essential for both the acute and longer-term effects of GLP1RA on food intake and body weight. While there is no doubt that direct application of GLP1RA directly into other structures in the brain can produce metabolic effects [21,45] more compelling data are required to determine the relative importance of these structures for clinically relevant, GLP1RA-mediated weight loss in obese models. The latter may become apparent with wider adoption of longer treatment regimens.

### 3.4 GIPR expression in the brain

**Figure 4:**
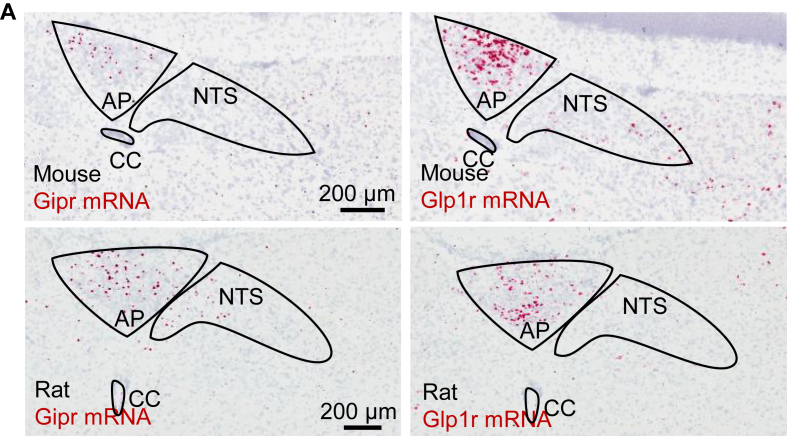
***Gipr* and *Glp1r* mRNA expression in the mouse and rat hindbrain.** Labelled RNAscope analysis showing *Gipr* mRNA expression (red) in the mouse (top left panel) and rat (bottom left panel) is concentrated in the area postrema (AP) and sparse in the nucleus of the solitary tract (NTS). *Glp1r* mRNA expression (red) in the mouse (top right panel) and rat (bottom right panel) is also concentrated in the AP. Scale bars are 200 μm. AP, area postrema; CC, central canal, NTS, nucleus of the solitary tract.

Both *in situ* hybridisation histology and radioactive GIP binding on rat brain, report GIPR in the AP and some forebrain structures, including the olfactory bulb, cortex and lateral septum, but neither the NTS nor hypothalamus [26,27]. The generation of a transgenic *Gipr*-Cre mouse line has suggested GIPR is much more widely expressed in the mouse forebrain and brainstem, including in a number of hypothalamic nuclei and the NTS, in addition to the AP [28,29]. Chemogenetic activation of mouse *Gipr*-Cre neurons in either the hypothalamus or brainstem reduce body weight, though it is not certain that there is selective activation of native GIPR neurons alone [28,29]. For example, the strong anorexia and conditioned taste avoidance caused by activation of mouse *Gipr*-Cre neurons is unexpected due to published evidence that GIPRA reduce aversion [13,14]. To investigate a possible explanation, we re-evaluated *Gipr* and *Glp1r* mRNA expression in areas of the mouse and rat brain using RNAscope (Suppl Fig. 4A for validation of probes in pancreas). Generally, the distribution patterns in the hypothalamus and brainstem were the same for mouse and rat, though more cells were detected in the rat using this method (Figure 4A). The only significant expression of *Gipr* mRNA in either species was in the AP, which fits with previous reports (Figure 4A) [26,27]. There are only scattered *Gipr*-positive cells in the NTS, also confirming recently published papers [13,14]. We also confirmed that *Gipr* and *Glp1r* are in distinct cells within the AP/NTS (Suppl Fig. 4B). As expected *Glp1r* is expressed in the brainstem PBN and in the hypothalamic ARH, dorsomedial nucleus (DMH), PVH and LHA. Importantly, no *Gipr*-positive cells were recorded in these areas (Suppl Fig. 4C–G). The exception were some *Gipr*-positive cells close to the third ventricle in the posterior hypothalamus, adjacent to the DMH and ARH. Both the mouse and the rat have a small group of *Gipr* mRNA-positive neurons confined to the medial CeA (Suppl Fig. 4I and J). Finally, *Glp1r*, but not *Gipr*, is strongly expressed in the SFO, a CVO within the forebrain (Suppl Fig. 4H).

**Figure 5:**
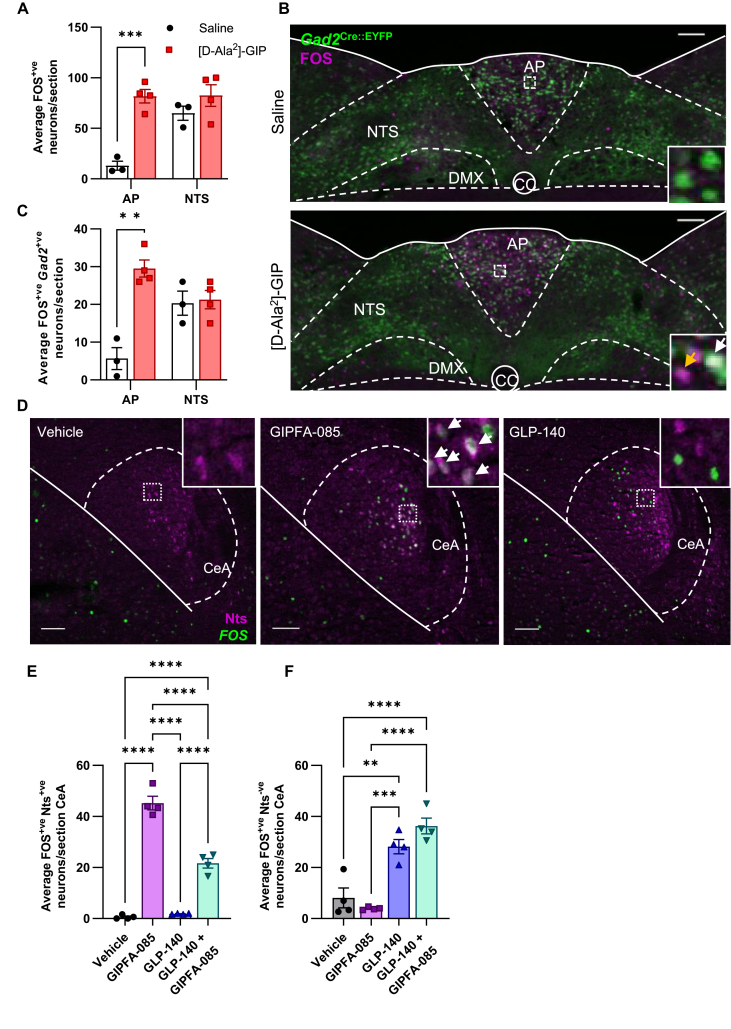
**GIPRAs activate specific neurons in the AP and CeA.** (A) FOS activity is increased in the area postrema (AP), but not in the nucleus of the solitary tract (NTS) of mice treated with an acute IP injection of [D-Ala^2^]-GIP (100 μg kg^−1^; red squares; n = 4) compared to saline (black circles; n = 3; ∗∗∗p < 0.001, Student's unpaired t-test). (B) Representative photomicrograph of GFP (green) and FOS (magenta) immunohistochemical staining in the hindbrain of *Gad2*^Cre::EYFP^ mice (white arrow shows co-localisation of FOS and GFP, yellow arrow shows FOS alone). (C) FOS activity is increased in *Gad2*^Cre::EYFP^-positive cells in the AP, but not in the NTS of mice treated with [D-Ala^2^]-GIP (red squares) compared to vehicle (black circles; n = 4 per group; ∗∗p < 0.01, Student's unpaired t-test). (D) Representative photomicrograph of Nts (magenta) and FOS (green) immunohistochemical staining in the central amygdala (CeA) of vehicle, GIPFA-085 and GLP-140 treated mice (300 nmol kg^−1^ and 30 nmol kg^−1^, respectively). (E) FOS activity is increased in Nts-positive neurons in the CeA after acute treatment with GIPFA-085 (purple squares; white arrows show co-localisation) and a combination of GLP-140 and GIPFA-085 (green inverted triangles), but not GLP-140 alone (blue triangles), compared to vehicle (black circles). In comparison, (F) FOS activity is increased in Nts-negative neurons in the CeA after treatment with GLP-140 alone (blue triangles) and a combination of GLP-140 and GIPFA-085 (green inverted triangles), but not GIPFA-085 alone (purple squares), compared to vehicle (n = 4 per group; ∗∗p < 0.01, ∗∗∗p < 0.001, ∗∗∗∗p < 0.0001, one-way ANOVA followed by Tukey's *post hoc* multiple comparison test). Scale bars are 100 μm. Inserts: digital zoom depicting cell bodies and nuclei. Data are presented as mean ± SEM. AP, area postrema; CeA, central amygdala nucleus; CC, central canal; DMX, dorsal motor nucleus of the vagus nerve; NTS, nucleus of the solitary tract.

We interpret these results, together with the brain penetrance and FOS activity data provided here and elsewhere, to infer that the GIPR agonism of both single- and dual-receptor molecules is largely dependent on GIPR neurons within the AP, which contain GABA [13,14]. We confirmed this by giving [D-Ala^2^]-GIP to *Gad2*^Cre::EYFP^ mice. [D-Ala^2^]-GIP caused a significant activation of neurons in the AP, but not in the NTS (Figure 5A–C). A limited number of *Gad2*^Cre::EYFP^ mice were also given an injection of the retrograde tracer, Fluorogold, into the PBN. Triple-label immunohistochemistry demonstrated that [D-Ala^2^]-GIP-responsive GABAergic neurons do not project to the PBN (Suppl Fig. 5A–B), the main second order region for AP neurons. This supports previous studies that suggest that the main target for GIP in the brainstem is GABAergic neurons in the AP that inhibit the local GLP1R/CCK/GFRAL neurons projecting directly to the PBN [13,14,36]. Importantly, these GLP1R-responsive neurons (either their collaterals or a sub-population) also send a projection to the NTS [25]. Currently, we do not know if GIPR/GABA neurons inhibit this or any other, independent pathway.

### 3.5 GIP activates specific neurons in the CeA

A noteworthy result from the unbiased activity mapping presented above was that the CeA expressed FOS after injection of either GIPRA or GLP1RA alone, and that there was an incremental increase with the combination treatment (Figure 2; Suppl Fig. 2). In a separate experiment, we found that systemic injection of the mono-GIPR agonist, GIPFA-085, alone activated a population of CeA cells containing neurotensin (Nts^CeA^), (Figure 5D–F). By comparison, GLP-140 activated only Nts-negative cells (Figure 5F). Interestingly, co-adminstration of the two agonists caused a significant reduction in the proportion of activated Nts-positive neurons in the CeA when compared with GIPFA-085 alone (Figure 5E). Currently, we have no evidence that systemically administered incretin can access the CeA - as shown above using fluorescently labelled TZP and GLP1RAs [[20], [21], [22]] - though we cannot discount the possibility entirely. As noted, there is a small group of *Gipr* mRNA-positive neurons confined to the medial CeA (Suppl Fig. 4I and J), whereas the induction of FOS by GIPFA-085 is mostly in the lateral CeA. Nonetheless, regardless of how GIPRA recruit Nts^CeA^ neurons, this result fits with published data that demonstrate complex interactions between local inhibitory neurons which potentially lead to reduced appetitive output from the CeA [[46], [47], [48]], an effect that could contribute to therapeutic action of dual GLP1RA/GIPRAs. The CeA is a complex structure with several sub-regions and many cell types present. It consists of GABAergic striatal-like neurons that are involved in both appetitive and defensive behaviours [46,47]. Multiple pathways from the primary sensing cells in the AP to the CeA can be envisaged. For example, we have proposed GLP1RA activates GLP1R/CCK/GFRAL neurons projecting directly to the PBN, and thence to PKCδ neurons primarily in the lateral portion of the CeA [12,13,25]. This is part of a well-established pathway resulting in anorexia and aversion [24,[49], [50], [51]]. PKCδ neurons inhibit other GABAergic neurons in the CeA, leading to disinhibition of the PSTN (another region displaying FOS after GLP1RA; Figure 2) [48]. The route involved in mediating the GIPR-activation of Nts^CeA^ neurons is to be resolved.

### 3.6 Nts^CeA^ neurons have a complex role

**Figure 6:**
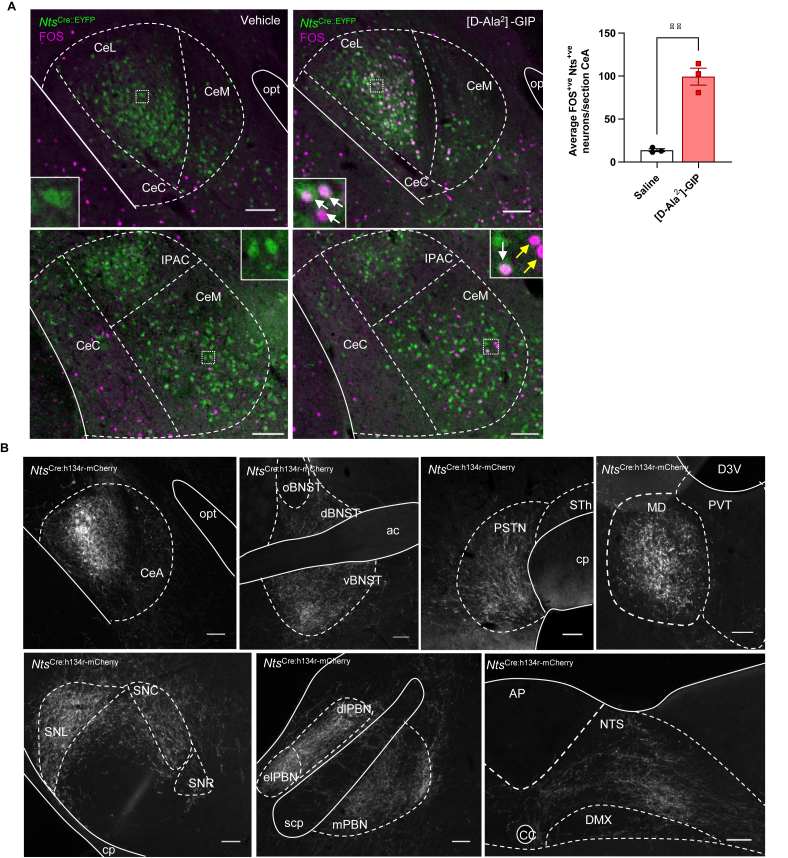
**Neurotensin-expressing neurons in the CeA are activated by GIP and project to areas elsewhere in the brain.** (A) Left and middle panels: Representative immunohistochemical images of GFP (green) and FOS (magenta) immunohistochemical staining in the CeA (top: caudal CeA; bottom: rostral CeA) of *Nts*^Cre::EYFP^ mice after IP injection with saline or [D-Ala^2^]- GIP (100 μg kg^−1^). Inserts: digital zoom depicting cell bodies and nuclei. White arrows show co-localisation of FOS and GFP, yellow arrows show FOS alone. Right panel: FOS activity is increased in *Nts*-expressing neurons in the CeA after injection of [D-Ala^2^]-GIP (red squares) compared to vehicle (black circles) (n = 3 per group; ∗∗p < 0.01, Student's unpaired t-test). Data are presented as mean ± SEM. (B) Representative immunohistochemical images from *Nts*^Cre^ mice injected unilaterally with AAV8-hSyn-DIO-ChR2(h134r)-mCherry as an anterograde tracer (*Nts*^Cre:h134r-mCherry^). Top row, left to right: Injection site in the central amygdala (CeA; opt, optic tract), projections to the ventral bed nucleus of the stria terminalis (vBSNT; ac, anterior commissure; d and o, dorsal and oval BNST), parasubthalamic nucleus (PSTN; cp, cerebral peduncle; STh, subthalamic nucleus) and medial dorsal thalamic nucleus (MD; D3V, dorsal third ventricle; PVT, paraventricular nucleus of the thalamus). Bottom row, left to right: Projections to the lateral substantia nigra (SNL; C and R, compact and reticular), parabrachial nucleus (PBN; dl, el and m, dorsolateral, exterolateral and medial; scp, superior cerebellar peduncle) and nucleus of the solitary tract (NTS; AP, area postrema; cc, central canal; DMX, dorsal motor nucleus of the vagus nerve). Scale bars are 100 μm.

Nts^CeA^ neurons have been classified generally as being appetitive, as their activation sustains self-stimulation [46]. They also are activated by food intake, while designer caspase-mediated ablation of Nts^CeA^ neurons significantly reduces alcohol consumption, without changes in general fluid consumption or body weight [24]. Thus, we tested a transgenic *Nts*^Cre^ model by crossing it with a fluorescent reporter mouse (R26R-EYFP) and by injecting reporter-expressing offspring with either vehicle or the short-acting [D-Ala^2^] -GIP. We were able to confirm that, although *Nts*^Cre:EYFP^ neurons are quite widespread, GIPRA activates neurons selectively in the CeA (Figure 6A). Next, we targeted these cells with an anterograde tracer, AAV-DIO-ChR2(h134r)-mCherry. Though, with this approach, we cannot restrict virus injection only to GIPRA-responsive cells, we could demonstrate widespread projections of Nts^CeA^ neurons, most prominently to the BNST, PSTN, medial dorsal thalamic nucleus, substantia nigra, PBN and NTS (Figure 6B). Thus, the Nts^CeA^ neurons may include GABAergic neurons that project to the PSTN, which may themselves be inhibited by GLP1A-activated PKCδ^CeA^ neurons, thus disinhibiting the PSTN and reducing feeding behaviour [46,48]. Or they may modulate ascending inputs from the NTS and PBN. Torruella-Suárez et al. [52] previously demonstrated that stimulation of the Nts^CeA^ projection to the PBN is reinforcing and increases consumption of palatable fluids, without affecting overall food intake. There is much current debate in the literature about whether it is more advantageous to antagonise rather than agonise GIPR receptors to produce the best outcome for body weight regulation [18,37,[53], [54], [55]]. However, currently, the only prescribed dual incretin receptor drug is TZP, which is an agonist at both GIP and GLP1R.

**Figure 7:**
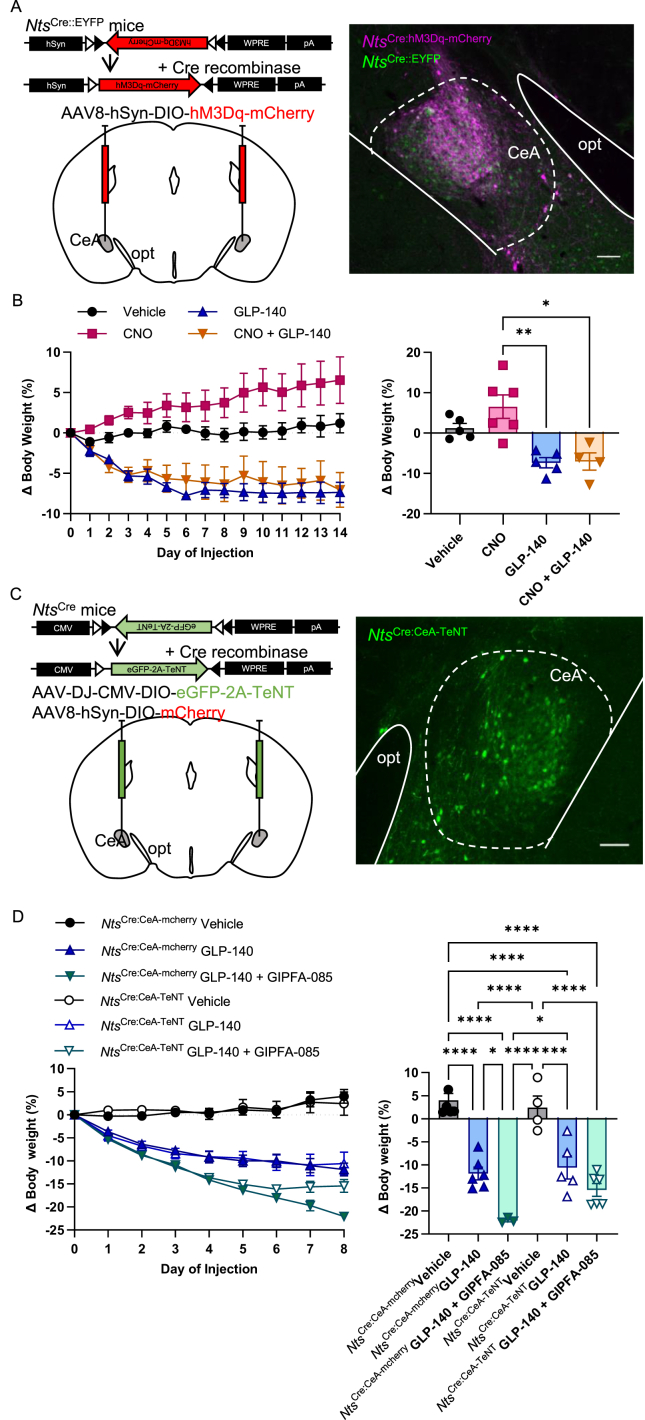
**Genetic manipulation of neurotensin-expressing neurons in the CeA.** (A) Schematic illustrating bilateral injection of AAV8-hSyn-DIO-hM3Dq-mCherry into the CeA of *Nts*^Cre::EYFP^ mice (left panel). Representative immunohistochemical image of AAV8-hSyn-DIO-hM3Dq-mCherry (*Nts*^Cre:hM3Dq-mCherry^; magenta) injection into the CeA of *Nts*^Cre::EYFP^ (green) mice (right panel). (B) Body-weight change in *Nts*^Cre:hM3Dq-mCherry^ mice following treatment with vehicle (black circles; n = 5), CNO (1 mg kg^−1^; pink squares; n = 6), GLP-140 (10 nmol kg^−1^; blue triangles; n = 5) or a combination of CNO and GLP-140 (orange inverted triangles; n = 4). ∗p < 0.05, ∗∗p < 0.01, two-way ANOVA followed by Tukey's *post hoc* multiple comparison tests. (C) Schematic illustrating bilateral injection of AAV-DJ-CMV-DIO-eGFP-2A-TeNT or AAV8-hSyn-DIO-mCherry as control into CeA of *Nts*^Cre^ mice (left panel). Representative immunohistochemical image of AAV-DJ-CMV-DIO-eGFP-2A-TeNT injection into the CeA of *Nts*^Cre^ mice (green *Nts*^Cre:CeA−TeNT^, green; right panel). (B) Body weight change in *Nts*^Cre:CeA-mCherry^ control and *Nts*^Cre:CeA−TeNT^ mice following treatment with vehicle (black closed triangles; n = 6 and black open circles; n = 4, respectively), GLP-140 (30 nmol kg^−1^; blue closed triangles; n = 6 and blue open triangles; n = 5, respectively) or a combination of GIPFA-085 and GLP-140 (green closed inverted triangles; n = 3 and green open inverted triangles; n = 8, respectively). ∗p < 0.05, ∗∗∗p < 0.001, ∗∗∗∗p < 0.0001 by two-way ANOVA followed by Tukey's *post hoc* multiple comparison tests. Scale bars are 100 μm. Data are presented as mean ± SEM.

To investigate the potential relevance of this small population of Nts^CeA^ neurons that appear responsive to systemically administered incretin receptor agonists, we carried out gain- and loss-of-function studies. First, we bilaterally transfected Nts^CeA^ neurons with a virus encoding an excitatory designer receptor, AAV-DIO-hM3Dq-mCherry, before placing the mice onto high-energy diet (Figure 7A). Subsequently, the DIO *Nts*^Cre^ mice were given daily subcutaneous injections of vehicle, GLP-140, the designer drug CNO, or GLP-140 and CNO in combination. In this experiment, the vehicle control mice maintained a steady body weight. GLP-140 caused a 10% decrease in body weight after 5 days, and this was sustained during 14-day treatment (Figure 7B). As expected, the GLP-140 mice reduced food intake rapidly (Suppl Fig. 6A). By contrast, there was a small but steady increase (approximate 5% by day 14) in body weight with CNO alone, concomitant with increased food intake. This finding is congruent with other studies in lean mice that have shown Nts^CeA^ neurons are appetitive [46]. The two treatments together followed the same body-weight pattern as GLP-140 alone, indicating that activation of this separate pathway is capable of overcoming the effect of stimulating Nts^CeA^ neurons, and may reflect local inhibition within the CeA.

Finally, we sought to functionally disable the Nts^CeA^ neurons, by injecting *Nts*^Cre^ mice bilaterally into the CeA with a virus encoding the light chain of tetanus toxin, AAV-DIO-eGFP-2A-TeNT (Figure 7C). Tetanus toxin does not kill the neurons but prevents them from releasing their transmitters. Alternatively, another set of *Nts*^Cre^ mice were injected bilaterally instead with a control virus, AAV-DIO-mCherry. Mice from each set were treated either with vehicle, GLP-140 or a combination of GLP-140 and GIPFA-085. As published previously, the reduction in weight caused by GLP1RA administration alone is enhanced by co-administration of GIPRA (Figure 7D) [7,15,16]. By comparison, this interaction is lost in *Nts*^Cre^ mice in which Nts^CeA^ neurons are disabled with tetanus toxin. Mice administered with GLP-140 or a combination GLP-140 and GIPFA-085 exhibited a rapid reduction in food intake (Suppl Fig. 6B). Together, these experiments suggest a complex role for Nts^CeA^ neurons in the effects of incretin drugs and in affecting the synergy noted by dual agonism.

### 3.7 Limitation of study

As with other published data looking at brain penetrance of other GLP1RA [[20], [21], [22]], our study recorded limited distribution after a single injection of fluorescently labelled TZP. Repeated injection or infusions might reveal different patterns and/or greater penetrance. Likewise, our whole-brain mapping experiments also involved single injections. The pattern of brain activity might be different with repeat injections and could potentially be assessed using alternative transcriptional markers [56]. There is a clear mismatch between different approaches to locate GIPR within the rodent brain. Our own RNAscope data matches that of other published data [26,27], demonstrating a limited distribution of both mRNA expression and ligand binding, but with a clear “hotspot” in the AP. Our data also matches the limited brain activity induced by administration of GIP, which is also backed up by functional experiments [13,14]. By comparison, the distribution of *Gipr*-Cre cells in one model is widespread within the brain, including in the NTS and hypothalamus [28,29], where we see only scattered, if any, *Gipr* mRNA-positive cells. Although our evidence points towards the AP as being the primary target for systemic incretin agonists to affect body weight in obese rodents, we look forward to validation of other direct targets. Here we have concentrated on identifying downstream targets involved in the synergy between GLP1RA and GIPRA. Recent interest has highlighted the potential for GIPR antagonism to have similar potential for weight loss, which may involve different cellular targets [[53], [54], [55]]. Finally, it is difficult to isolate experimentally the Nts^CeA^ neurons that are activated specifically by GIPRA, because there are many neurotensin cells surrounding them within the complex CeA structure. Thus, although we attempted FOS-TRAP to isolate these cells [57], background recombination made that experiment impossible to interpret. Alternative approaches for the future might include an exhaustive study of each of the projections of these neurons, perhaps using retrogradely transported designer receptors.

## CRediT authorship contribution statement

**Claire H. Feetham:** Writing – review & editing, Writing – original draft, Methodology, Investigation, Formal analysis, Data curation. **Minrong Ai:** Writing – review & editing, Writing – original draft, Methodology, Investigation, Formal analysis, Data curation. **Isabella Culotta:** Writing – review & editing, Methodology, Investigation, Formal analysis. **Alessia Costa:** Writing – review & editing, Methodology, Investigation, Formal analysis. **Jenna Hunter:** Writing – review & editing, Investigation. **Robert A. Brown:** Writing - review & editing, Methodology, Investigation, Formal analysis. **Tamer Coskun:** Writing – review & editing, Writing – original draft, Supervision, Conceptualization. **Paul J. Emmerson:** Writing – review & editing, Writing – original draft, Supervision. **Giuseppe D'Agostino:** Writing – review & editing, Writing – original draft, Supervision, Funding acquisition, Conceptualization. **Simon M. Luckman:** Writing – review & editing, Writing – original draft, Supervision, Funding acquisition, Conceptualization.

## Funding

This work was funded by Biotechnology and Biological Sciences Research Council grants to SML (BB/S008098/1, BB/Z516405/1) and Medical Research Council grants to SML/GD’A (MR/T032669/1; MR/Y014707/1) and GD’A (MR/P009824/2), and an additional direct contribution from Eli Lilly. AC was supported for part of this project by a travel grant from the Italian Society of Pharmacology and a fellowship from the Veronesi Foundation (Italy).

## Declaration of competing interest

The authors declare the following financial interests/personal relationships which may be considered as potential competing interests: Simon Luckman and Giuseppe D'Agostino reports financial support was provided by Eli Lilly and Company. Paul Emmerson, Robert Brown, Tamer Coskun and Minrong Ai reports a relationship with Eli Lilly and Company that includes: employment. If there are other authors, they declare that they have no known competing financial interests or personal relationships that could have appeared to influence the work reported in this paper.

## Data Availability

Data will be made available on request.

## LIST OF ABBREVIATIONS

AAV: adeno-associated virus
ac: anterior commissure
ACB: nucleus accumbens
ANOVA: analysis of variance
AP: area postrema
ARH: arcuate nucleus of the hypothalamus
BNST: bed nucleus of the stria terminalis
CC: central canal
CCK: cholecystokinin
CeA: central amygdala
CeL: lateral portion of the central amygdala
CNO: clozapine N-oxide
CP: caudoputamen
Cre: Cre recombinase
CVO: circumventricular organ
D3V: dorsal third ventricle
DAPI: 4′,6-diamidino-2-phenylindole
DIO: diet-induced obesity
DMH: dorsomedial nucleus of the hypothalamus
DMX: dorsal motor nucleus of vagus
EYFP: enhanced yellow fluorescent peptide
flox: flanked by loxP sites
FITC: fluorescein isothiocyanate
GABA: γ-amino butyric acid
GFRAL: GDNF-family receptor α-like receptor
GIPRA: glucose-dependent insulinotropic polypeptide receptor agonists
GLP1RA: glucagon-like peptide-1 receptor agonists
HED: high-energy diet
IP: intraperitoneal
LHA: lateral hypothalamic area
MD: medial dorsal nucleus of the thalamus
ME: median eminence
Nts: neurotensin
NTS: nucleus of the tractus solitarius
OV/OVLT: organum vasculosum of the lamina terminalis
PBN: parabrachial nucleus
PBS: phosphate-buffered saline
PKCδ: protein kinase Cδ
PSTN: parasubthalamic nucleus
PPG: preproglucagon
PVH: paraventricular nucleus of the hypothalamus
PVT: paraventricular nucleus of the thalamus
SC: sub-cutaneous
scp: superior cerebellar peduncle
SEM: standard error of mean
SEMA: semaglutide
SFO: subfornical organ
TeNT: tetanus toxin
TZP: tirzepatide
VEH: vehicle
VTA: ventral tegmental area
wt: wild-type
