# Supplementary material for: Downstream interaction by glucagon-like peptide-1 and glucose-dependent insulinotropic polypeptide agonism is required for synergistic effects on body weight
Source: Mol Metab. 2025 Jul 16;99:102214. doi: 10.1016/j.molmet.2025.102214 (PMC12336652; doi:10.1016/j.molmet.2025.102214)
Supplement: Multimedia component 1 [file mmc1.pdf]

# APPENDIX A. SUPPLEMENTARY DATA

## Supplementary Figure 1

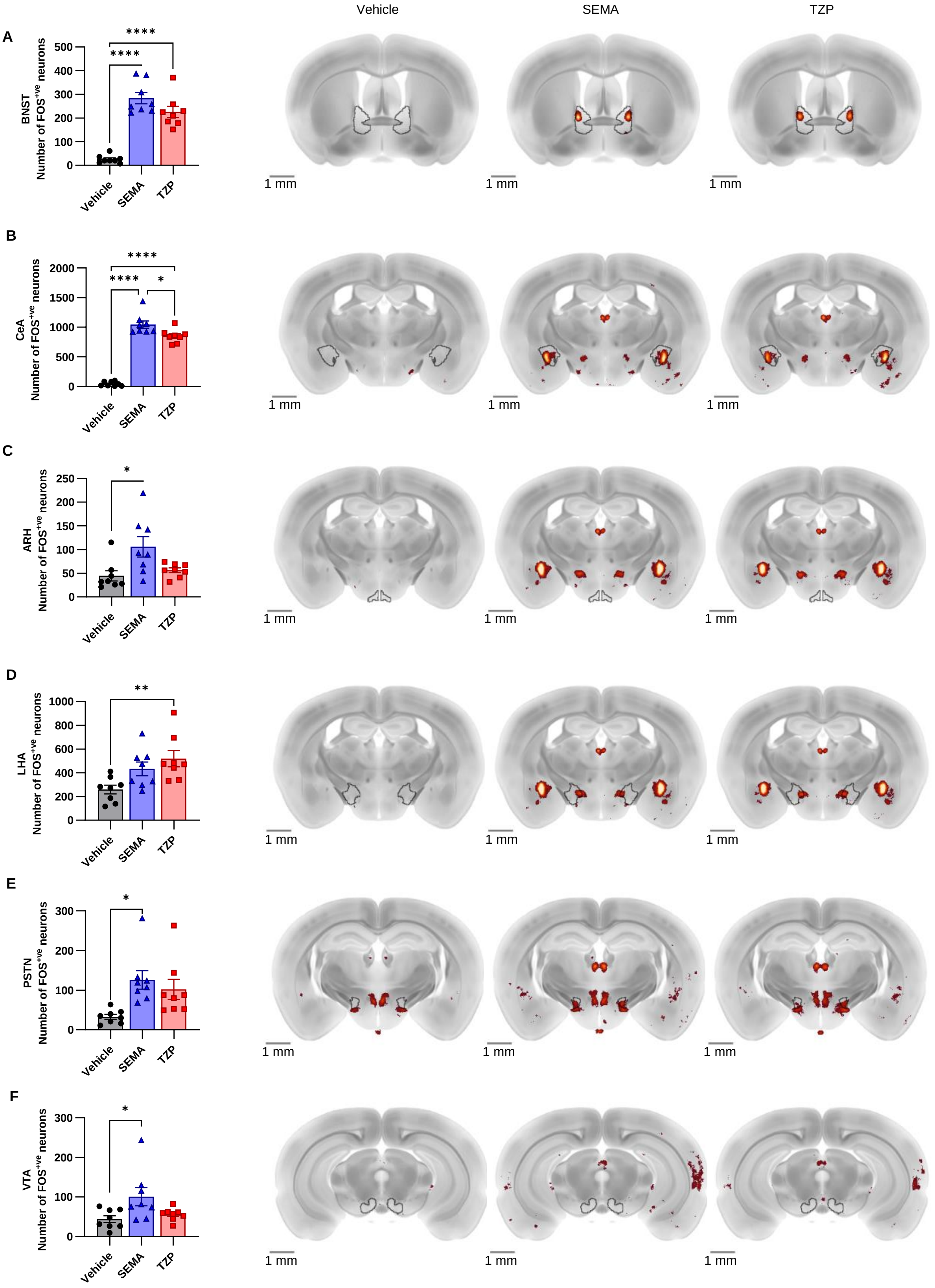

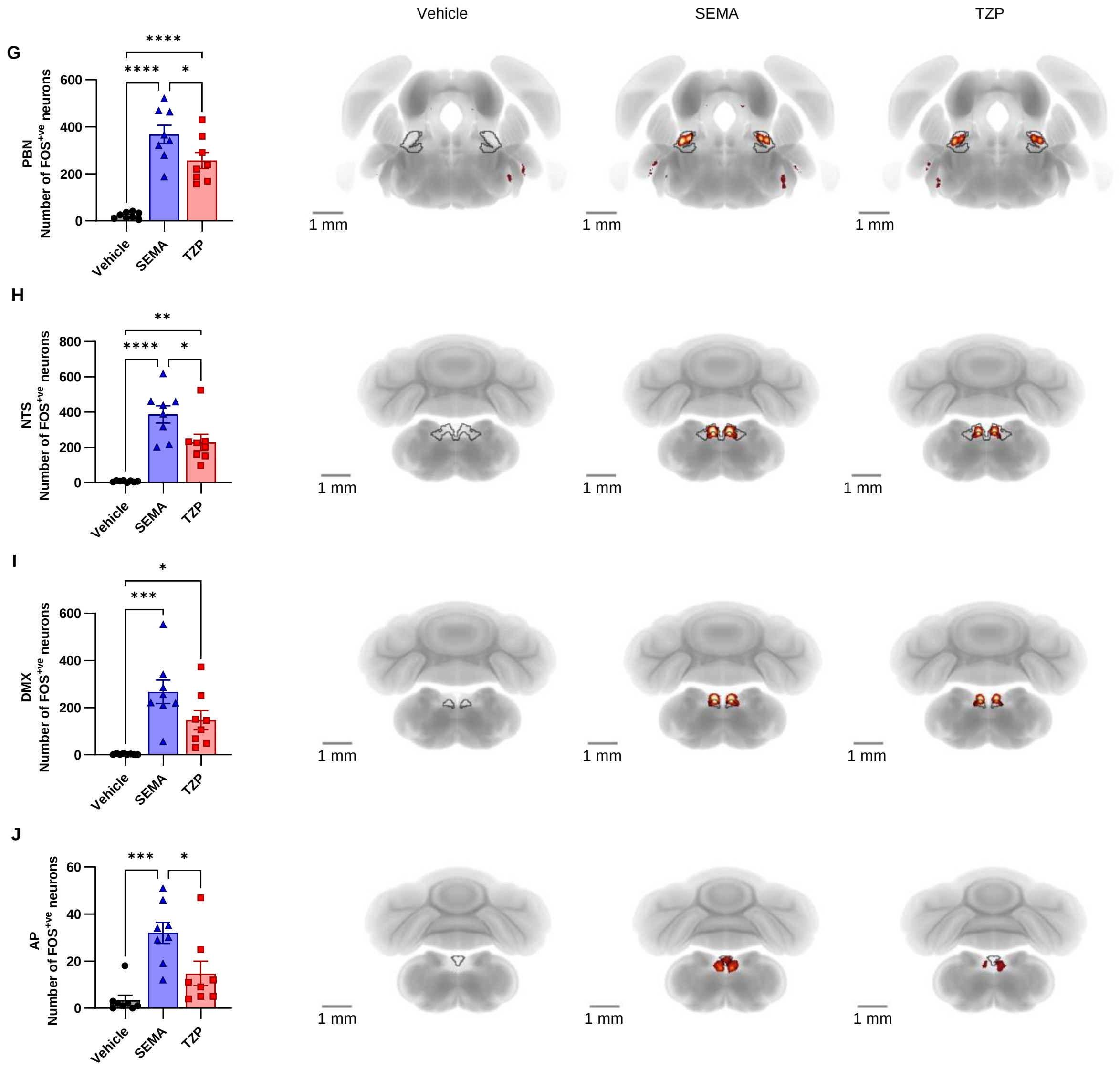


**Supplementary Figure 1** Analysis of FOS activity (left panel) following treatment with vehicle (black circles), SEMA (30 nmol kg$^{-1}$; blue triangles) and TZP (30 nmol kg$^{-1}$; red squares; n = 8 per group) and the corresponding density heatmaps of FOS group averages in glow superimposed onto coronal sections from the Gubra brain atlas (right panel) in the (A) BNST , (B) CeA, (C) ARH, (D) LHA, (E) PSTN, (F) VTA, (G) PBN, (H) NTS, (I) DMX, (J) AP. *p<0.05, **p<0.01, ***p<0.001, ****p<0.0001 by one-way ANOVA followed by Tukey's *post hoc* multiple comparison tests. Scale bars are 1 mm. Data are presented as mean ± SEM. AP, area postrema; ARH, arcuate nucleus of the hypothalamus; BNST, bed nucleus of the stria terminalis; CeA, central amygdala nucleus; DMH, dorsomedial nucleus of the hypothalamus; DMX, dorsal motor nucleus of the vagus nerve; LHA, lateral hypothalamic area; NTS, nucleus of the solitary tract; PBN, parabrachial nucleus; PSTN, parasubthalamic nucleus; VTA, ventral tegmental area.

## Supplementary Figure 2

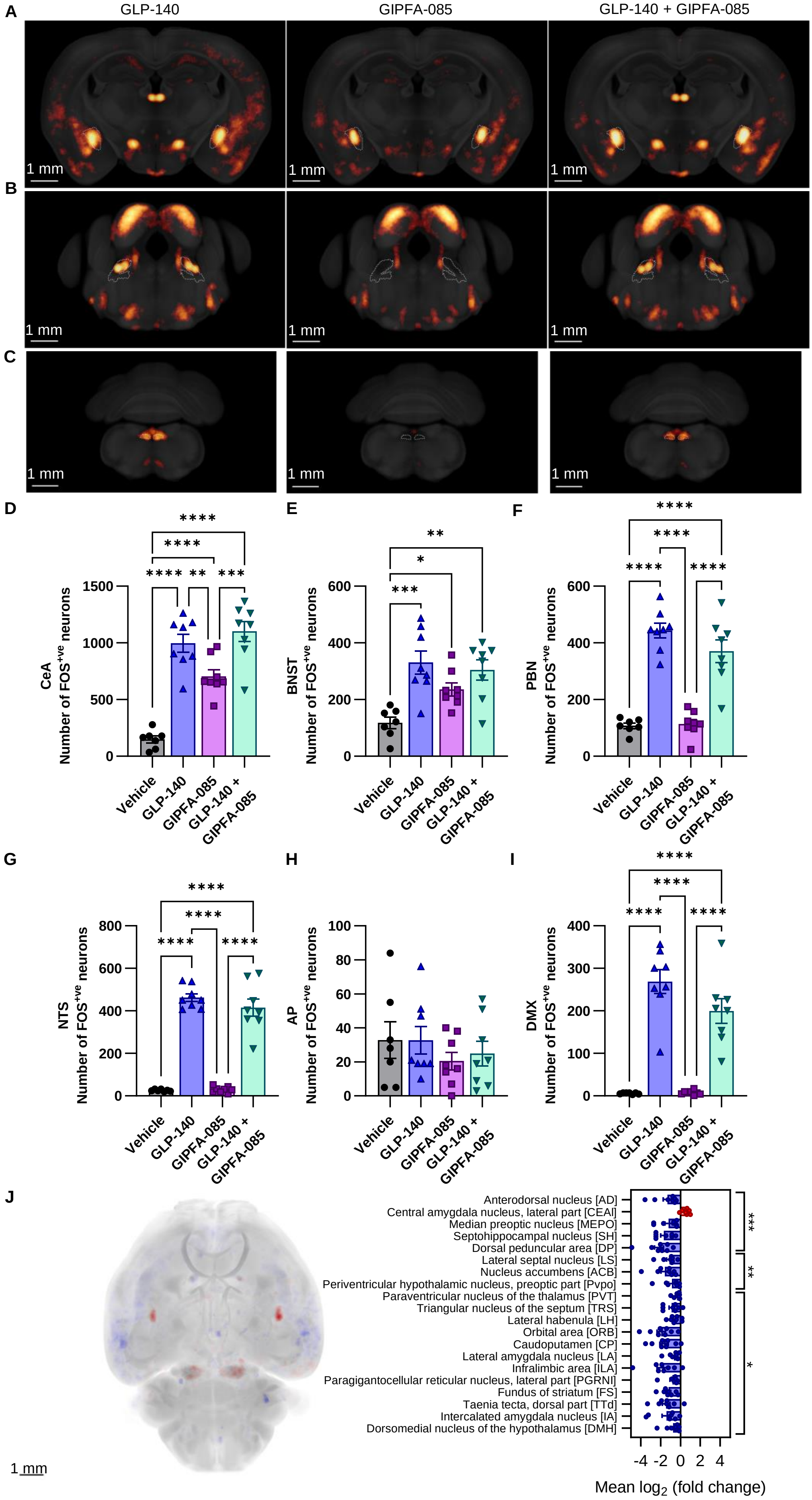

**Supplementary Figure 2** Density heatmaps of FOS group averages superimposed onto coronal sections from the Gubra brain atlas in mice treated with GLP-140 (30 nmol $kg^{-1}$), GIPFA-085 (300 nmol $kg^{-1}$) or a combination of GLP-140 and GIPFA-085 showing the (A) CeA, (B) PBN and (C) hindbrain. FOS activity following treatment with vehicle (black circles; n = 7), GLP-140 (blue triangles; n = 8), GIPFA-085 (purple squares; n = 8), and combination of GLP-140 and GIPFA-085 (green inverted triangles) in the (D) CeA, (E) BNST, (F) PBN, (G) NTS, (H) AP and (I) DMX. **p<0.01, ***p<0.001, ****p<0.0001 by one-way ANOVA followed by Tukey's *post hoc* multiple comparison tests. (J) Top 20 brain regions where FOS activity is changed in the combination GLP-140 and GIPFA-085 treatment group compared to GLP-140 alone ((mean $log_2$ (fold change); n = 8 per group, *p<0.05, **p<0.01, ***p<0.001, Students unpaired *t*-test). Areas with increased FOS activity are shown in red and areas with reduced FOS activity in blue. Scale bars are 1 mm. Data are presented as mean ± SEM. AP, area postrema; BNST, bed nucleus of the stria terminalis; CeA, central amygdala nucleus; DMX, dorsal motor nucleus of the vagus; NTS, nucleus of the solitary tract; PBN, parabrachial nucleus.

## Supplementary Figure 3

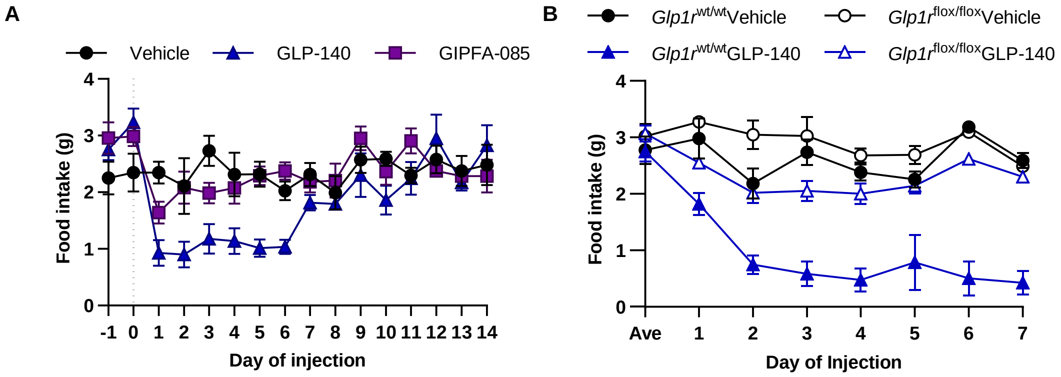


**Supplementary Figure 3** (A) Food intake in C57BL/6J mice following treatment with vehicle (black circles), GLP-140 (blue triangles) and GIPFA-085 (purple squares; n = 5 per group). (B) Food intake in *Glpr1r*$^{wt/wt}$ and *Glpr1r*$^{flox/flox:Cre\text{-}TdTomato}$ mice following treatment with vehicle (black closed circles; n = 5 and black open circles; n = 3, respectively) and GLP-140 (blue closed triangles; n = 5 and blue open triangles; n = 4, respectively). Data are presented as mean ± SEM.

# Supplementary Figure 4

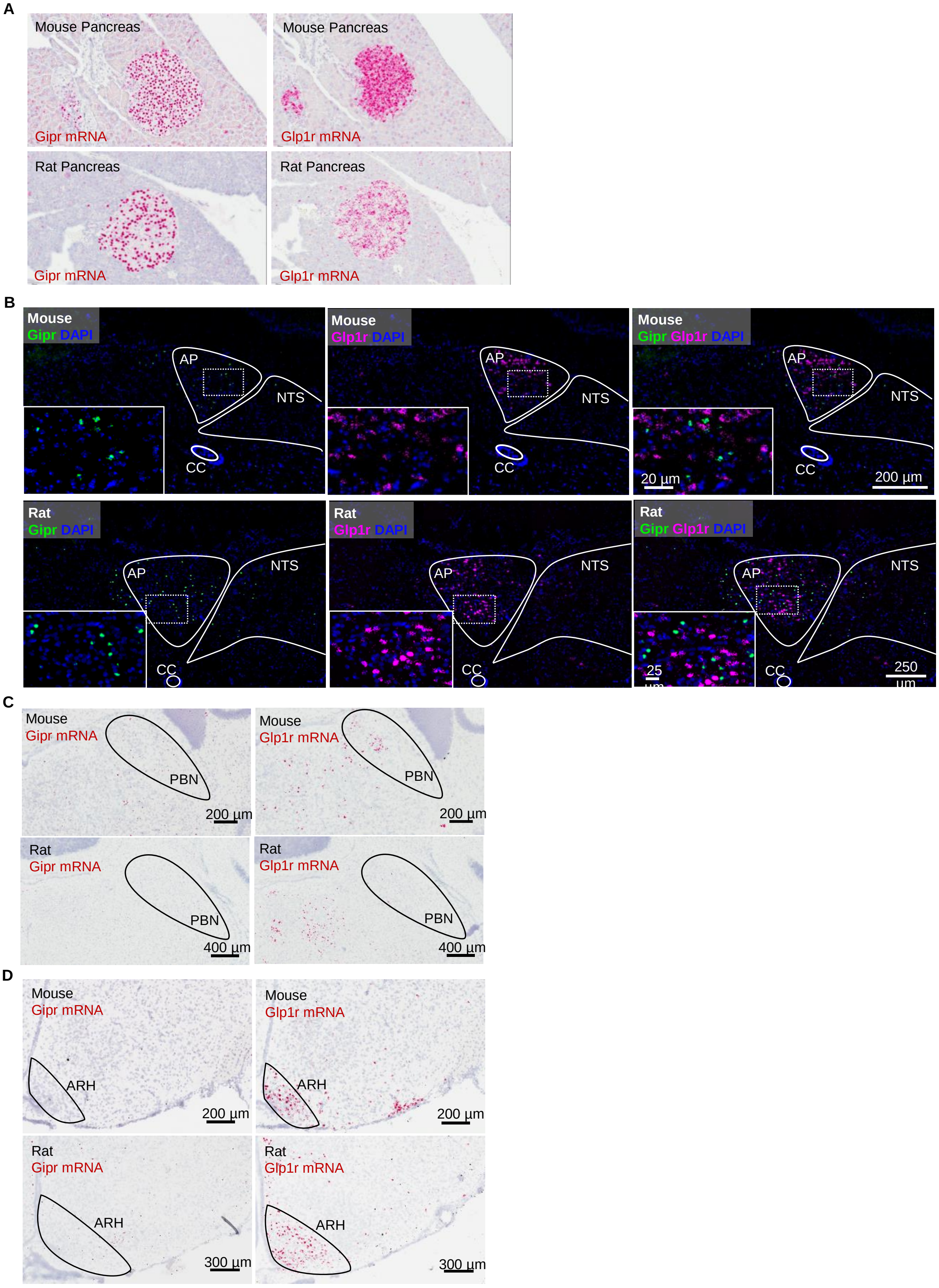

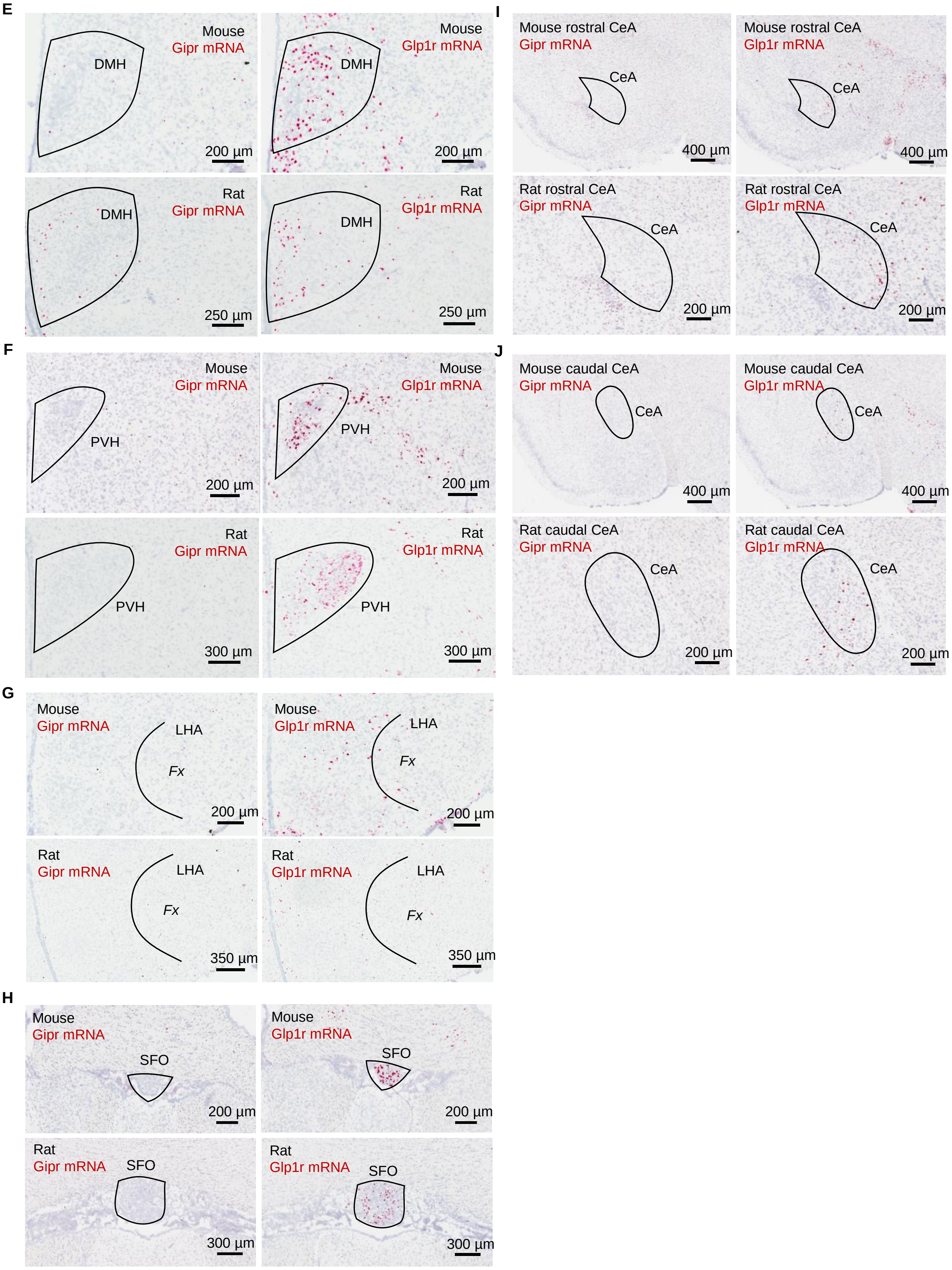


**Supplementary Figure 4** (A) Representative labelled RNAscope images showing *Gipr* (left panel) and *Glp1r* (right panel) mRNA expression (shown in red) in the pancreas as a control tissue in the mouse (top panel) and rat (bottom panel). (B) Representative RNAscope images from mouse (top panel) and rat (bottom panel) showing *Gipr* mRNA (green) does not overlap with *Glp1r* mRNA (magenta) in the hindbrain of either species (DAPI in blue). Scale bars are 200 µm in mouse and 250 µm in rat. Inserts: higher magnification depicting mRNA staining and DAPI (blue), scale bars are 20 µm in mouse and 25 µm in rat. Representative labelled RNAscope images showing *Gipr* (left

panel) and *Glp1r* (right panel) mRNA expression (shown in red) in the mouse (top panel) and rat (bottom panel) (C) PBN, (D) ARH, (E) DMH, (F) PVH, (G) LHA, (H) SFO, (I) rostral CeA and (J) caudal CeA. Scale bars are indicated on the corresponding images. AP, area postrema; ARH, arcuate nucleus of the hypothalamus; CC, central canal; CeA, central amygdala nucleus; DMH, dorsomedial nucleus of the hypothalamus; LHA, lateral hypothalamic area; NTS, nucleus of the solitary tract; PVH, paraventricular nucleus of the hypothalamus; SFO, subfornical organ.

## Supplementary Figure 5

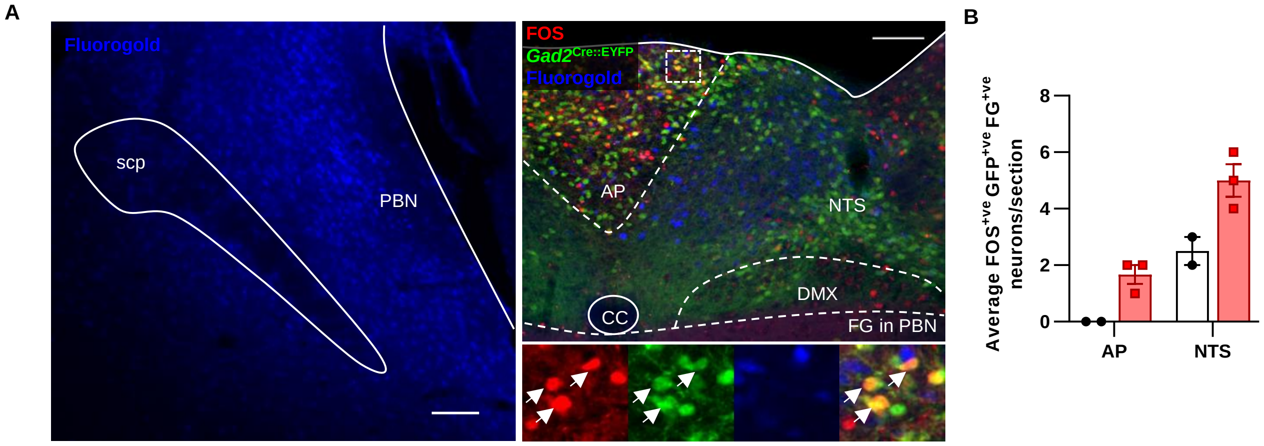


**Supplementary Figure 5** (A) Representative photomicrograph of unilateral injection of the retrograde tracer, FluoroGold (FG; blue), into the parabrachial nucleus (PBN) of *Gad2*$^{Cre::EYFP}$ mice (left panel). Triple-fluorescence staining showing [D-Ala$^2$]-GIP-activated Gad2 neurons in the nucleus of the solitary tract (NTS) do not project to the parabrachial nucleus (PBN; right panel). FOS (red), GPF (green) and FG (blue). Inserts: digital zoom depicting cell bodies and nuclei. White arrow shows co-localisation of FOS and GFP. Scale bars are 100 µm. (B) Only a small number of FG-positive, GFP-positive neurons are activated following vehicle (n = 2) or [D-Ala$^2$]-GIP treatment (n = 3). Data are presented as mean ± SEM. AP, area postrema; CC, central canal; DMX, dorsal motor nucleus of the vagus nerve; PBN, parabrachial nucleus; NTS, nucleus of the solitary tract; scp, superior cerebellar peduncle**.**

## Supplementary Figure 6

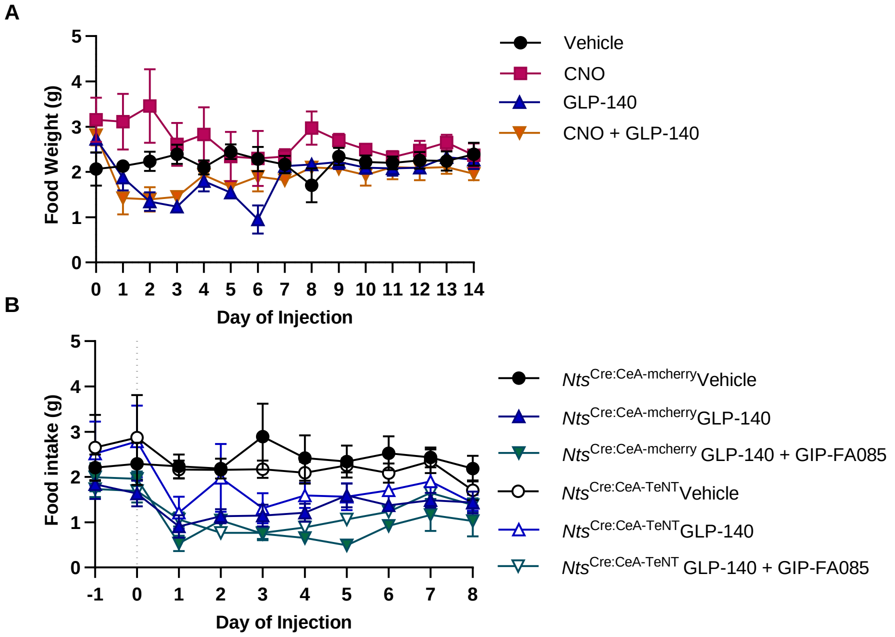


**Supplementary Figure 6** (A) Food intake in *Nts*$^{Cre:hM3Dq\text{-}mCherry}$ mice following treatment with vehicle (black circles; n = 5), CNO (1 mg kg$^{-1}$; pink squares; n = 6), GLP-140 (10 nmol kg$^{-1}$; blue triangles; n = 5) or a combination of CNO and GLP-140 (orange inverted triangles; n = 4). (B) Food intake in *Nts*$^{Cre:CeA\text{-}mCherry}$ control and *Nts*$^{Cre:CeA\text{-}TeNT}$ mice following treatment with vehicle (black closed circles; n = 6 and black open circles; n = 4, respectively), GLP-140 (30 nmol kg$^{-1}$; blue closed triangles; n = 6 and blue open triangles; n = 5, respectively) or a combination of GIPFA-085 and GLP-140 (green closed inverted triangles; n = 3 and green open inverted triangles; n = 8, respectively). Data are presented as mean ± SEM.

**Supplementary Video 1** Representative whole-brain distribution of fluorescently labelled TZP-AF647. TZP-AF647 fluorescent signal has been subtracted from vehicle treatment and is shown in glow and background autofluorescence is grey.

**Supplementary Video 2** Representative whole-brain FOS staining in a vehicle-treated C57BL/6J mouse. FOS fluorescent signal is shown in glow and background autofluorescence is grey.

**Supplementary Video 3** Representative whole-brain FOS staining in a SEMA-treated C57BL/6J mouse. FOS fluorescent signal is shown in glow and background autofluorescence is grey.

**Supplementary Video 4** Representative whole-brain FOS staining in a TZP-treated C57BL/6J mouse. FOS fluorescent signal is shown in glow and background autofluorescence is grey.
